# Targeting Complement Pathways in Polytrauma- and Sepsis-Induced Multiple-Organ Dysfunction

**DOI:** 10.3389/fimmu.2019.00543

**Published:** 2019-03-21

**Authors:** Ebru Karasu, Bo Nilsson, Jörg Köhl, John D. Lambris, Markus Huber-Lang

**Affiliations:** ^1^Institute for Clinical and Experimental Trauma-Immunology, University Hospital of Ulm, Ulm, Germany; ^2^Department of Immunology, Genetics and Pathology (IGP), Laboratory C5:3, Uppsala University, Uppsala, Sweden; ^3^Institute for Systemic Inflammation Research (ISEF), University of Lübeck, Lübeck, Germany; ^4^Division of Immunobiology, Cincinnati Children's Hospital, Cincinnati, OH, United States; ^5^Department of Pathology & Laboratory Medicine, University of Pennsylvania School of Medicine, Philadelphia, PA, United States

**Keywords:** trauma, sepsis, hemorrhagic shock, MODS, complement activation, complement dysregulation, complement therapeutics, clinical trial

## Abstract

Exposure to traumatic or infectious insults results in a rapid activation of the complement cascade as major fluid defense system of innate immunity. The complement system acts as a master alarm system during the molecular danger response after trauma and significantly contributes to the clearance of DAMPs and PAMPs. However, depending on the origin and extent of the damaged macro- and micro -milieu, the complement system can also be either excessively activated or inhibited. In both cases, this can lead to a maladaptive immune response and subsequent multiple cellular and organ dysfunction. The arsenal of complement-specific drugs offers promising strategies for various critical conditions after trauma, hemorrhagic shock, sepsis, and multiple organ failure. The imbalanced immune response needs to be detected in a rational and real-time manner before the translational therapeutic potential of these drugs can be fully utilized. Overall, the temporal-spatial complement response after tissue trauma and during sepsis remains somewhat enigmatic and demands a clinical triad: reliable tissue damage assessment, complement activation monitoring, and potent complement targeting to highly specific rebalance the fluid phase innate immune response.

## Introduction

Complement activation as a major innate defense strategy occurs early after trauma, hemorrhagic shock and during sepsis in both the experimental and clinical settings (1–6). A recent comparison of severe trauma and septic patients in an intensive care unit (ICU) showed that during sepsis, excessive activation of the complement cascade is detectable as evidenced by significantly enhanced systemic C3a concentrations, whereas during trauma, complement activation is also existent but less pronounced (7). In trauma, rapid consumption of key complement components such as C3 or C5 seems to be the primary mechanisms (7) whereas in septic conditions, consumption of complement factors may occur later (if at all), secondary to the activation. Hyper-activated and consumed defense systems including the complement and coagulation cascade can result in imbalanced immune responses, impaired clearance of tissue debris and pathogens, dysregulated coagulation, perfusion disturbances, changes in tissue and cellular microenvironment, and barrier dysfunction. All these alterations culminate in multiple signaling-, cellular-, and organ dysfunction (8, 9). Although highly specific complement inhibitors are available, translation of these observations into therapeutic strategies remains challenging and requires differential considerations (10). The immune response to damaged and infected tissue is not mono-dimensional; instead it comprises various compartmentalized responses and organ specific outcomes (11, 12). For example, in experimental sepsis there is a loss of C5aR expression on neutrophils whereas C5aR expression on various organs is significantly enhanced (13). Thus, the complement reaction to danger associated molecular patterns (DAMPs) and pathogen-associated molecular patterns (PAMPs) needs to be reliably determined before any specific therapeutic intervention can be applied in the clinical setting of severe sterile or infectious insults. Furthermore, underlying triggers and mechanisms of the multiple organ dysfunction syndrome (MODS) need to be detected and further explored. Several hypotheses exist about the initiators and drivers of MODS. Main contributors seem to be barrier failure (9), electrophysiological alterations (14), microcirculatory disturbances, inflammation-induced cell dysfunction, protein alterations and microbiome shifts (15). Hibernation seems to be a contributing factor in the development of MODS, which allows the cell to shut down energy consuming functional efforts and therefore to preserve the cellular morphology (16). Although MODS can be induced by distinct injuries and infectious insults, once established, it seems to follow common pathways. For the individual and also the society, MODS remains a major burden with a high lethality rate and a high socio-economic impact and therefore requires an improved understanding, comprehensive mechanistic insights and basic as well as clinical research efforts (17).

## Polytrauma-Induced MODS—Role of Complement

**Figure 1:**
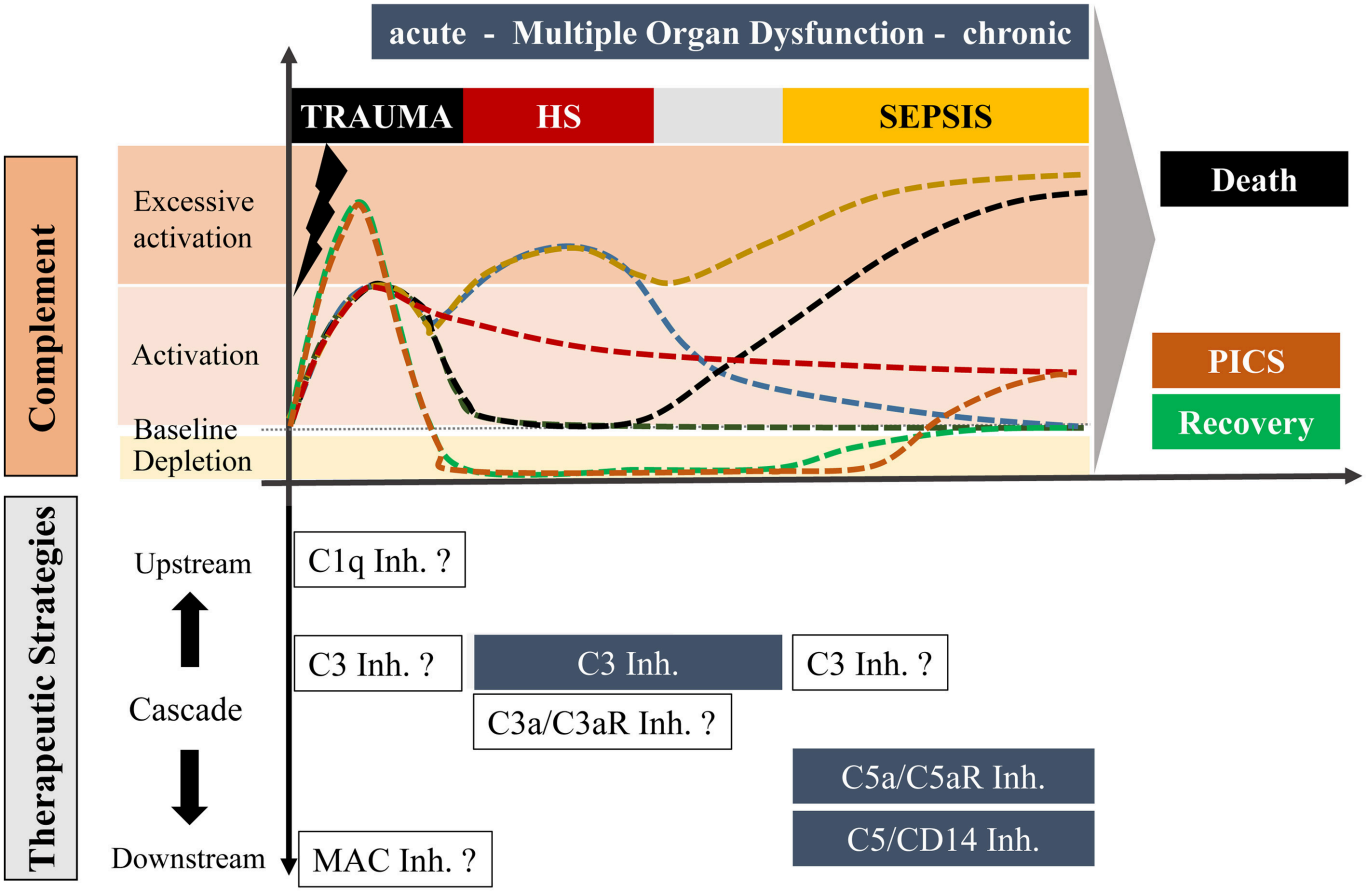
Overview of potential complement responses after trauma with or without hemorrhagic shock and sepsis leading to either death or recovery or “Persistent Inflammation, Immunosuppression and Catabolism Syndrome” (PICS; with a constitutively active and chronic complement state) and potential complement-targeting strategies, respectively. The upper scaling represents the temporal course of complement activation with possible scenarios. Under optimal conditions, trauma induces an acute complement activation which follows a rapid decline to a physiological state with recovery (dark green). Alternatively, complement activity can also be maintained over a long period which may be associated with PICS (red). In an unfavorable state, the acute complement activation could be followed by secondary complication/s including sepsis leading to a complement hyperactivation and death (black). Trauma with an additional hemorrhagic shock can cause excessive complement activation, which can resolve in a physiological recovery. However, an additional hemorrhagic shock with hyperactivated complement can also result in a life-threatening sepsis phase (yellow). Trauma can further cause an acute complement hyperactivation with a rapid consumption of complement which may finally be resolved in a recovery phase (green) or to complement activation at the later stage leading to chronic inflammation (orange). The downwards orientated scaling includes possible therapeutic strategies of distinct stages of complement after trauma, hemorrhagic shock and sepsis. Blue highlighted boxes include therapeutic options which already revealed beneficial effects in pre-clinical studies. Non-highlighted boxes include possible strategies which need to be investigated in future experimental approaches.

Polytrauma comprises life-threatening multiple injuries that activate innate and adaptive immunity with multidimensional consequences for the host (9, 18). Although some reduction in the frequency of MODS after polytrauma has been noted in the last decades, it still remains a major cause of death after severe trauma (19). Within minutes after polytrauma there is a significant increase in circulating complement activation products such as C3a, C5a, and sC5b-9 and a drop in complement hemolytic activity (2–4, 6, 20) (Figure 1). Of note, an enhanced C3a/C3 ratio in plasma early after trauma was prognostic for lethal outcome (6). Another study showed enhanced C3a levels as an indicator for MODS (20). Activation via its amplification by the alternative pathway is observed early after trauma measured by Bb plasma levels, which was additionally correlated with injury severity and the development of organ failure such as acute lung injury and acute renal failure (3). The underlying mechanisms, however, are still elusive. For example, C3a may have a direct pathophysiological impact on the lungs as an “engine” of multiple organ failure. C3a alters not only the microvascular and airway tonus (21) but induces direct pro-inflammatory effects (22) which may contribute to perfusion disturbances and cellular dysfunction. In a murine model of blunt thorax trauma, we have found enhanced C3 and heme-oxygenase-1 (HMOX1) transcriptional expression levels in the lungs early after injury (23). A recent analyses of 81 polytrauma patients also revealed an enhanced expression of HMOX1, which was associated with septic complications (24). In this context, it is noteworthy that HMOX1 is downregulated in leukemic leukocytes by C3a and C5a resulting in an enhanced cellular mobility and infectious complications (25), indicating some interaction between HMOX1 and complement activation processes. Clinical trials, evaluating the effect of targeting at the C3or C3a/C3aR are lacking. Although in a different context, a recent study showed the potential of HMOX1 to protect endothelial cells against heme-mediated complement activation. Heme activates the alternative complement pathway and also up-regulates the cyto-protective and stress-response gene HMOX1 in an organ-specific manner. While it was highly upregulated in endothelial cells of large vessels, it was poorly upregulated in the renal endothelium, which makes the renal endothelium more vulnerably for complement over-activation and showed stronger C3 deposition (26).

On the C5 level, the generated anaphylatoxin C5a is a potent chemoattractant that enhances surface expression of intercellular adhesion molecules on the endothelium, and thereby effectively recruits inflammatory cells to the injured site (27). The migrated cells of the first line of defense can sense, phagocyte and clear damaged tissue and induced repair processes (9). In a recent polytrauma study, leukocytes with low C5-expression on day 1 after trauma correlated with an increased risk for the development of nosocomial infections during the later course (28). The corresponding C5a receptors, C5aR1 and C5aR2, are down-regulated on leukocytes early after trauma (29), something that has been proposed as a sign of enhanced risk for infectious complications (30). Of note, no clinical trial has been proposed or designed in regard to polytrauma using downstream modulation principals of the complement cascade, e.g., modulation of the membrane attack complex (MAC). This might represent an interesting pharmaceutical target, especially since significant amounts of sC5-9 are generated early after polytrauma (2).

## Hemorrhagic-Shock-Induced MODS—Role of Complement

Hemorrhagic shock is a condition of disturbed tissue perfusion, resulting in the inadequate delivery of oxygen and nutrients and inadequate clearance of waste products, all of which are vital for regular cellular function. The involvement of complement is complex.

Complement activation products have been reported to directly or indirectly alter the vascular tonus. A C3a-analog peptide was able to cause pulmonary artery constriction in a thromboxan-dependent manner (31). Similarly, C5a caused transient vasoconstriction of an isolated pulmonary artery, which was dependent on the integrity of the endothelium (32). In other reports, C5a caused a leukocyte-dependent vasospasm and triggered thromboembolic events by C5a-induced release of thromboxane-like eicosanoids (33). In line with this observation, C5a is involved in the pathogenesis of acute kidney injury in hemorrhagic shock by reducing renal blood flow by ~ 30% and the glomerular filtration rate by ~ 45%, respectively (34). Of note, when either a leukotriene antagonist was applied or neutrophils were depleted, the C5a-induced reduction in renal perfusion was not observed indicating a leukotriene-dependent (34). In contrast, systemic application of C5a in rabbits induced a reversible drop in systemic arterial pressure (MAP) and a drop in central venous pressure (CVP), decreased cardiac output (CO), and neutropenia (35). In regard to coronary artery vasotonus, conflicting data have been reported for C5a both, vasoconstrictory (36) and vasodilatory effects (37). Other studies concluded that anaphylatoxins alter the vascular tonus with a high variability depending on the localization and pre-exposure to other mediators (38). Taken together, the available data suggest that complement activation changes tissue perfusion by macro- and micro-hemodynamic effects, especially when a shock is already established.

Another central pathomechanistic driver of hemorrhagic shock is the ischemia/reperfusion injury. In this regard, several studies have shown direct activation of complement with a significant increase in MBL and C3 on the endothelium by hypoxic and reperfusion conditions (39). Of note, clinically and experimentally, hemorrhagic shock is a major driver of further organ damage and development of coagulopathy, endotheliopathy, barrier failure, immune dysfunction and MODS after polytrauma (40–43). The close relation of hemorrhagic shock and complement activation reflected by C3 consumption has been previously modeled in baboons where cobra venom factor (CVF) was injected to activate and deplete C3 before a subsequent hemorrhagic shock was induced (20). In pigs, hemorrhagic shock resulted in a drop of CH50 during the hypovolemic phase and also during early resuscitation with enhanced plasma levels of C5a and early detection of endotoxin in the blood, which contributes to MODS (44). A proteomic approach using a rodent hemorrhagic shock model revealed a few alterations of the lymphatic fluid (“toxic lymph”), which included significantly enhanced C3 precursor protein concentrations (45). Since the “toxic lymph” is considered as an important pathophysiological mechanism in the development of adult respiratory distress syndrome and MODS, these findings indicate C3 as a promising target for immune modulatory approaches (Figure 1).

## Sepsis-Induced MODS—Role of Complement

Sepsis was long considered as systemic inflammatory response syndrome with evidence of pathogenic microorganisms (46). In contrast, the new definitions describe sepsis rather as organ dysfunction induced by a dysregulated host response to infection (47). This paradigm shift (48) may also change the focus of therapeutic strategies toward support of organ functions, far beyond the established eradication of the pathogens. However, it remains somehow enigmatic what “dysregulation” of the host response means or if the “inadequacy” of the host response is central for development and progression of sepsis.

Multiple experimental sepsis studies have emphasized the detrimental effects of excessive complement activation for the host (Figure 1) (49, 50). This can be considered as a “complement paradox” since complement *per se* is central to the innate immune defense against invading microorganisms. However, in the context of MODS development caused by sepsis, complement activation seems to enhance rather than protecting against several organ dysfunctions, especially in the heart, lungs and kidneys, representing three central organs in MODS (51–53). The multi-organ gene expression profiles in experimental sepsis seems to be either organ-specific, or common to more than one organ, or distinctly opposite in some organs (54). Furthermore, a balanced pro- and anti-inflammatory genetic response was observed and a differential gene expression for mediators responsible for preventing tissue damage, e.g., protease inhibitors, oxidant neutralizing enzymes, decoy receptors, and proteins which can protect tissue barriers (54). Concerning complement, pre-pro-complement C3 was highly expressed in all organs except in the brain during the whole course of sepsis (54). However, genetic deficiency of C3 resulted in significantly enhanced lethality in comparison to C3-sufficient mice most likely due to a loss of C3b-dependent opsonization of invaded pathogens (55). In contrast, a blockade of C5a by various strategies in sepsis models, e.g., by anti-C5a antibodies, C5aR antibodies, small peptide C5aR1 antagonists, C5a-neutralizing mirror-image (l-)aptamer C5a aptamers, was coherently protective against biochemical and histological evidence of MODS and in general improved survival of sepsis (53, 55–61). All these experimental results demonstrate that C5a-C5aR interaction is clearly involved in the pathogenesis of MODS during sepsis and represents an important therapeutic sepsis target when the novel definitions of sepsis are applied (Figure 1) (47).

In translation to the clinical setting, several non-human primate experiments and studies in humans are in line with the findings in the rodent sepsis model. Evidence of systemic complement activation with reduction of complement hemolytic activity, C3 depletion and enhanced levels of C3a and C5a and corresponding loss of C5aR on neutrophils have been described in several human studies (62–64). Of note, the reduction of C5aR1 and C5aR2 on neutrophils has been correlated with the occurrence of infectious complications in ICU patients (30) and sepsis-induced MODS (62, 65, 66). Since loss of C5aR1 and C5aR2 has been concurrently correlated to the sequential organ failure assessment (SOFA) score (65), a flow-based rapid testing, might have a bedside monitoring potential to predict infectious problems and MODS development.

## MODS—Pathomechansisms Caused by Multiple Complement Dysfunctions

**Figure 2:**
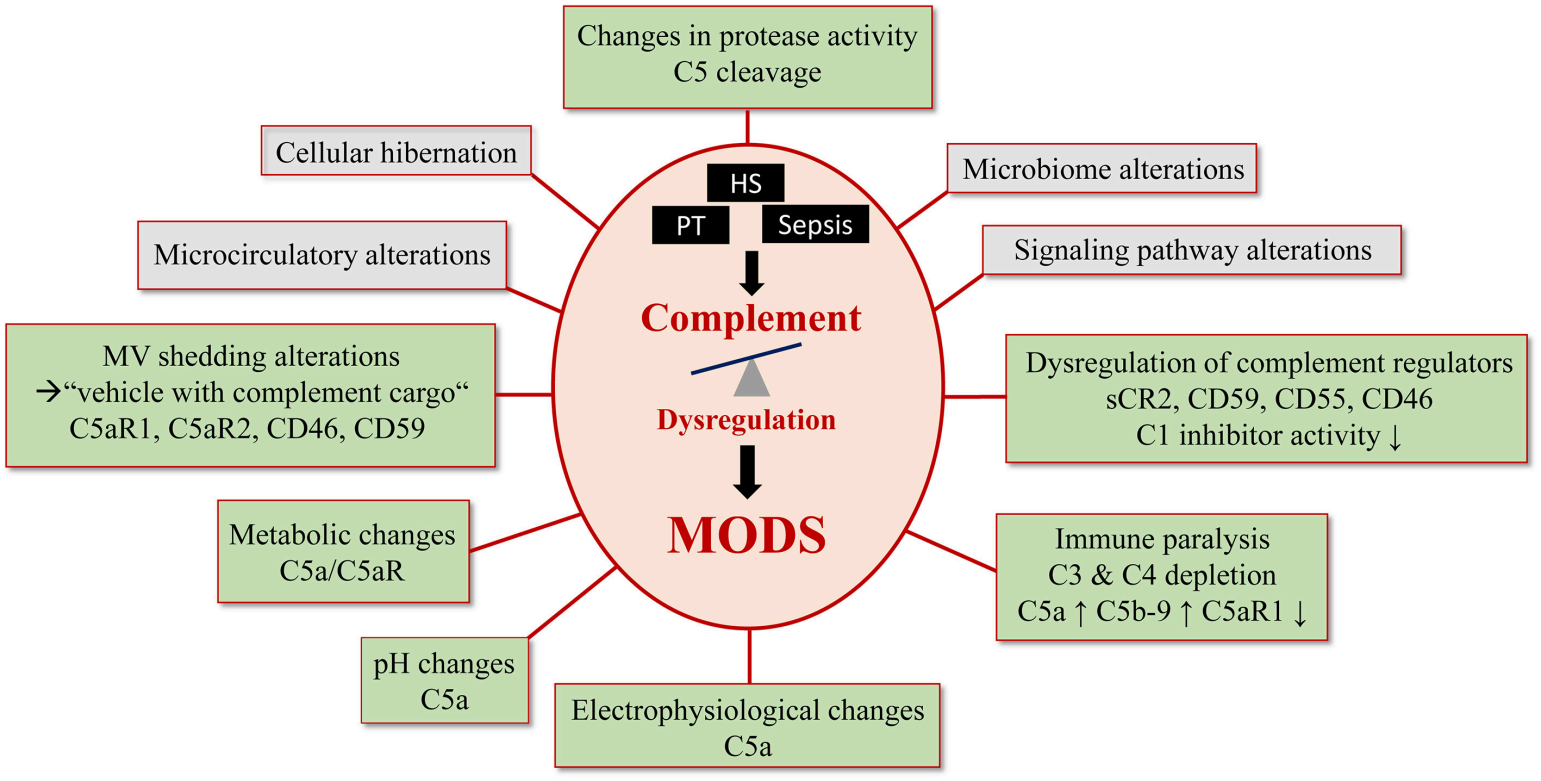
Complement- mediated pathomechanisms in MODS development. Polytrauma, sepsis and hemorrhagic shock cause a critical complement dysregulation, which causes dysfunction in multiple organs. This figure summarizes potential and known pathomechansims for MODS development caused by complement dysregulation. Established complement involvement is highlighted in light green and proposed complement involvement is highlighted in gray.

Several pathomechanisms contribute to the development of MODS, such as enhanced levels of DAMPs and PAMPs, reduced cytochrome P450 metabolism, macrophage activation syndrome, and cytokine-driven cellular dysfunction (67). Overall, it is clear that complement dysregulation contributes to MODS after trauma (Figure 2). Some of these aspects have been already mentioned and will be further discussed in this section.

### Immune Paralysis

Though severely injured patients receive modern ICU management, many of them show signs of immunosuppression known as persistent inflammation-immunosuppressive catabolism syndrome (PICS) (Figure 1) (68). Clinically, PICS patients suffer from persistent inflammation, immune suppression and protein catabolism, which can lead to recurrent nosocomial infections with sepsis, MODS and death (68). Severe immune suppression of the fluid-phase and cellular immune response has been proposed as “immune paralysis” of the host response to sterile and infectious insults. In a clinical case report, an inadequate response to infection with signs of systemic depletion of complement (dropping C3 and C4 levels) has been associated with the development of acute kidney injury and multiple organ failure in a 17-day old newborn (69). C5b-9 and C5a have been described as contributors to cell death, immune paralysis, cardiac dysfunction, and multiple organ failure (Figure 2) (49, 70). In support, a baboon model of *Escherichia coli* sepsis showed that blockade of C5 protected organs from “immune paralysis” and improved the sepsis survival rate (Table 1) (75).

**Table 1:** Most representative preclinical studies addressing trauma, HS and sepsis, respectively.

	**Model**	**Intervention**	**Outcome**	**References**
Trauma	Mouse TBI	CR2-fH: C3 deposition Inhibition	Less C3 deposition in brain; decreased microglia activation; less neuronal cell death	(71)
	Mouse TBI	C6 antisense oligonucleotide Inhibition of MAC formation Coversin (OMCI) C5 inhibition	Inhibition of C6: less cerebral MAC (up to 96%); inhibition of C6 synthesis (> 80%)	(72)
	Mouse TBI	CD59-2a-CRIg Terminal pathway (MAC) inhibition	Less axonal damage; enhanced neurological recovery; inhibition of C5: decreased MAC formation; improved neurological outcome	(73)
	Mouse TBI	CR2-CD59: Terminal pathway (MAC) inhibition CR2-Crry: all complement pathways inhibition CR2-fH: alternative complement pathway inhibition	Significantly improved chronic outcomes	(74)
Sepsis	Baboon septic shock	RA101295: C5 inhibition	Significantly improved survival; reduced inflammation and coagulopathy; significantly improved organ function	(75)
	Mouse meningococcal sepsis	PMX 205: C5aR antagonist	Protection from invasive meningococcal infection; enhanced mouse survival; ameliorated inflammatory cytokine response	(76)
	Piglets poly-microbial sepsis	Coversin (OMCI) C5 inhibition And Anti-CD14	Combined C5 and CD14 inhibition: significantly improved survival; significantly lower plasma sC5b-9 levels, which correlated with mortality	(77)
	Baboon septic shock	Compstatin: C3 inhibition	Significantly decreased procoagulant response; organ protection by significantly improved vascular barrier function; less leukocyte infiltration and cell death	(5)
HS	NHP HS	Comstatin C3 blockade	Protection of organ function; reduced intestinal edema; improved kidney function	(78)
	Swine trauma with HS	C1 inhibition	less TNF; less complement deposition[C3, C5 and C5b-9 (MAC)] in the small intestine and lungs; improvement of metabolic acidosis; less renal, intestinal, and lung tissue damage	(79)

*HS, hemorrhagic shock; MAC, membrane attack complex; NHP, non-human primate; TBI, traumatic brain injury; TNF, tumor necrosis factor*.

### Dysregulated Complement Regulators

Additional connection between MODS and signs of complementopathy are supported by the fact that soluble and membrane-bound regulators of complement activity show alterations after trauma, sepsis and hemorrhagic shock. It is well-established that severe tissue injury causes an excessive systemic intravascular activation of the complement system resulting in a loss over the control mechanisms (8). In this context, the soluble form of the complement receptor 2 (sCR2) was shown to be present after nerve injury in rodents (80). After polytrauma in humans, leukocyte expression profiles of the complement regulators (CRegs) CD55 (decay accelerating factor), CD59 (membrane attack complex inhibitor), CD46 (membrane cofactor protein), and CD35 (complement receptor 1) were cell-type- and time-dependently altered, reflecting manifestation of posttraumatic complementopathy (4, 29). Furthermore, an observational study including critically ill patients with multiple injuries and sepsis revealed that trauma patients with MODS have significantly lower C1-inhibitor activities (Figure 2) (7). A rare mutation in the complement regulatory gene factor H has been also implicated with a severe form of complement-mediated hemolytic uremic syndrome with multiple organ involvement, showing the general importance of complement regulation for organ (dys)functions (81).

### Alteration of Signaling Pathway Activity

Experimental studies showed an interplay of complement with specific signaling pathways including the activation of PKC, MAPKs, and ERK (82). In a non-traumatic renal damage model, NF-κB contributed to enhanced complement activation (83), while in a similar mouse model the C5a/C5aR axis activated MAPK signaling (84). During sepsis, the activation of MAPKs and Akt signaling was complement-induced (51). These mechanistic insights require further elaboration to identify other participating mediators downstream to complement activation in trauma and hemorrhagic shock.

### Changes in Protease Activity

Excessive systemic activation of proteases feed in the pathomechanisms of MODS and is known to create a vicious cycle of complement activation. Such proteases include immunoreactive trypsins and neutrophil elastase, which may directly interact with complement and have been extensively described in the case of polytrauma (9). A specific subtype of MODS has been introduced as thrombocytopenia-associated MODS, with low ADAMTS13 activity and defects in inhibitory complement regulators which may result in hyperactivation of coagulation and complement with resultant thrombosis (85). This form of MODS has been successfully treated by C5 blockade (eculizumab) and ADMTS13 reduction by plasma exchange (67). Furthermore, an unspecific and unregulated protease hyperactivity may directly cleave various complement components in a non-canonical manner, which represents a promising future research field for drug development. In this case, the intestine plays an important role in trauma and during shock, which can proceed to a proposed autodigestion phenomenon, since digestive enzymes from the pancreas are activated by enterokinases (86). Under physiological conditions, the auto-digestion is prevented by epithelial and mucosal barriers. However, during shock digestive peptides can pass the mucus and reach the epithelial cell membranes, where they disrupt junctional complexes, further activate complement and contribute barrier and organ dysfunction (87).

### Microcirculatory Alteration

Patients who succumbed to traumatic-hemorrhagic shock showed an impaired microcirculation for at least 72 h, which also was a reliable predictor for a high Sequential Organ Failure Assessment (SOFA) score (88). In an ischemia rat model, application of soluble complement receptor type 1 significantly improved microvascular perfusion in the liver assessed by *in vivo* microscopy, suggesting an essential role of complement in microcirculatory disorders (89). Activated complement with microvascular alterations can also cause a disruption of cellular barriers leading to edema formation in lung, brain, and liver (84, 90, 91). However, hypothetical complement-caused damage of specific tight junction molecules in specific organs needs still scientific verification. Such mechanisms of MODS development may also be supported by simultaneous activation of complement and coagulation e.g., by complement-dependent generation of thrombin, which is known to efficiently break down various endothelial barriers. Furthermore, complement hyperactivation has been associated with thrombotic microangiopathies causing endothelial cell activation and thrombus formation leading to hemolytic anemia, thrombocytopenia, and organ failure (91).

### Metabolic Changes

Besides microcirculatory problems, massive blood loss due to hemorrhage can cause acute circulatory failure resulting in lactic acidosis. Acute liver or renal dysfunctions are most often associated with decreased lactate clearance and a pronounced increase in blood lactate levels compared with shock patients without signs of liver or renal dysfunction (92). It is also known that shock states initiate a pronounced compensatory vasoconstriction, consequently leading to hypoxia and accumulation of metabolites associated with lactate acidosis and a low pH milieu (92, 93). In this context, complement dysfunction has a deleterious influence and correlates with a worse outcome after shock. Especially the anaphylatoxin C5a and its following signaling via the C5aR1 have been described to further enhance acidosis after septic shock. More precisely, *in vitro* stimulation of neutrophils from healthy donors with C5a causes C5aR1 signaling-mediated immunometabolic changes in neutrophils with an enhanced glucose uptake and enhanced glycolytic flux (Figure 2) (94). In turn, it can initiate an increased proton secretion and further lower the extracellular pH. In response to C5a exposure, intracellular pH in neutrophils significantly increases via activation of the Na+/H+ exchanger type 1 (NHE-1) leading to an impairment in neutrophil function *in vitro*. Supporting this evidence, neutrophils isolated from septic patients has been shown to exhibit an increased intracellular pH compared to healthy donors (94). These recent findings indicate that inflammatory processes with produced C5a is solely capable to significantly change the micro-milieu with lactate acidotic features even in the absence of an oxygen deficit. It only can be speculated on, that in the case of an additional oxygen deficit, caused by shock conditions and vascular dysfunction, the anaphylatoxins may even function as a metabolic switch toward lactate acidosis and MODS.

### Microbiome Alterations

The intestinal complement is suggested to cooperate in a close relationship with the gut microbiome (95). Therefore, another contributor to MODS seems to be the alteration of the gut microbiome after trauma/hemorrhagic shock and/or sepsis. The microbiome, which is described by the phylogenetic composition and taxon relative abundance of the bacteria, is significantly altered in the first 72 h after injury. This rapid change in intestinal microbiota represents a critical phenomenon that may influence outcomes after severe trauma (15). The composition of the microbiome may influence the activation/dysregulation of complement pathways or dysregulated complement may change the microbiome composition. In the skin, complement activation modulates the inflammatory milieu by changing the cutaneous microbiota (96). Considering MODS, a mechanistic explanation for complement-microbiome interaction still remains elusive and needs further research.

### Alterations in Microvesicle (MV) Shedding

Communication is essential for cellular homeostasis and vesicle shedding has been described to play a crucial role for maintaining proper immune cell function. Extracellular vesicle shedding is altered after inflammation and is considered as a crucial contributor to MODS after multiple injury and sepsis (97). Especially shedding of microvesicles (MV) have been implicated in several inflammatory conditions including sepsis and trauma. Increased amounts of CD41+ and CD31+/CD41–/AnnexinV– MV after sepsis, released by activated platelets and leukocytes have been shown to correlate with unfavorable outcomes (98).

Furthermore, MVs from patients with multiple organ failure support the coagulation system in triggering inflammation. In respect to complement, phosphatidylserine containing MVs also serve as platform for complement activation (99). Besides activation of complement on their surfaces, MVs represent transport vehicles sending complement as cargo to neighboring as well as cells in distance (99). Hence, MV from different cellular origins may contain complement receptors including C5aR but also CRegs such as CD46 and CD59, suggesting a putative role for complement activity (99). In accordance with this, loss of C5aR1, C5aR2, and C3aR on neutrophils after multiple injury was clinically present and was correlated to infectious complications and multiple organ dysfunction (Figure 2) (18).

### Electrophysiological Changes

Another concurrent theory of MODS addresses electrophysiological changes of the cellular membrane which have recently been found in neutrophils from septic pigs (14). The anaphylatoxin C5a was able to alter the membrane potential of neutrophils but not in the case of neutrophils during septic MODS where the electrophysiological response to C5a was somehow frozen (Figure 2) (14). Whether complement inhibitory strategies will stabilize the cellular membrane electrophysiology is currently under investigation.

### Cellular Hibernation

Trauma causes an alarming stress situation for the whole body with an extensive inflammatory response (9). Some studies indicate that trauma especially followed by additional sepsis causes hibernation in the cellular as well as fluid phase of innate immunity including the complement system. Reflecting the evolved habit of conserving physiological resources in the event of environmental stress, with inflammation ensues a similar mechanism where energy-consuming processes are shut down in the organism. Supporting this evidence, hibernation has been observed in the septic heart with ongoing metabolic changes including the upregulation of specific glucose transporters in cardiomyocytes (100). Besides its effects on metabolism, hibernation is known to affect various immune function including leukocyte migration, as well as adaptive immune responses and interestingly complement function, by lowering complement levels and reduced expression of C3 mRNA in the liver, which depicts a suggestive link post-shock. (101). However, another study demonstrated that hibernators are protected from shock-induced injury, inflammation, and organ function (102). Strikingly, arctic ground squirrels challenged with cardiac arrest or hemorrhagic shock showed no markers of organ damage, systemic inflammation, or loss of acid/base balance as indicated by a negative base excess. Neither reduced body temperature nor hibernation season are components of this protection, indicating still unknown mechanisms involved (102).

Unfortunately, no supporting data is available indicating that future research on the complement function during hibernation, especially following trauma and shock is needed. Further, the ability to induce a fully reversible state of immune suppression in humans by artificial hibernation might aid the treatment of several inflammatory and immune-mediated diseases.

## Targeting Complement Pathways in MODS

Despite improvements in trauma care, the morbidity and mortality of MODS remains very high.

Therefore, new therapeutic strategies are urgently needed. Since complement is critically involved in initiation and progression of MODS, targeting complement as well as molecules contributing to complement activation represent promising future clinical approaches. Other complement-associated inflammatory conditions already addressed such a targeting strategy. Above 20 complement-interfering drugs have been evaluated in clinical settings so far. A few of them received FDA approval for inflammatory indications including eculizumab targeting the terminal complement pathway starting from C5, which is currently used for the treatment of paroxysmal nocturnal hemoglobinuria (PNH) (103). As existing complement therapeutics cannot target every complement-driven disease status, it is a requisite to evaluate the different complement stages, their relevance, and target them in a disease-specific manner (10).

In the context of trauma, hemorrhagic shock and sepsis a few preclinical experimental studies focus on complement-targeting studies (Table 1). Besides complement activation products, other molecules including CRP, HMGB1, or mitochondrial DNA play crucial roles in contributing to complement dysregulation after sepsis and trauma, thus profoundly influencing secondary outcomes. Therefore, a rational approach seems to address molecules, which may further boost complement activity. Likewise, HMGB1 is a relevant danger molecule and complement has been described to regulate HMGB1 release from human neutrophils (104) such that, complement inhibition has proved to be protective in blast-induced acute lung injury in rats by ameliorating HMGB1-mediated inflammation (105).

### Targeting Complement After (poly)Trauma

In polytrauma patients, the only study of complement intervention early after trauma with RCT quality used C1 esterase inhibitor. However, this study has been terminated based on the heterogeneity of the patients (106). Experimentally, the majority of the complement targeting studies were performed in mouse traumatic brain injury (TBI) models. In this case, various interventions to inhibit complement activity, such as inhibitors for MAC formation and C3 deposition inhibitors have been applied, analyzed and reviewed elsewhere (9, 107, 108). To further clarify the impact of distinct complement activation pathways on neuroinflammation, a recent study compared the effect of complement inhibitors targeting upstream as well as downstream complement activity, respectively. This study brought into light that instead of downstream complement activation complexes, upstream complement activation products of the alternative pathway predominantly modulate and propagate chronic inflammation (74) (Table 1).

### Targeting Complement in Hemorrhagic Shock

Based on the deduced potential of complement inhibitors to improve micro-perfusion disturbances, ischemia/reperfusion injury and the inflammatory response during hemorrhagic shock, various shock models have been tested for possible benefits of complement interventions. In a rat model of pressure controlled hemorrhagic shock, complement depletion by CVF improved the recovery of the mean arterial pressure (MAP) post shock (109). In a pig model of pressure controlled hemorrhagic shock, application of a C1-inhibitor improved the functional performance and reduced C3 deposition on a multi-organ level including liver, small intestine and lungs (Table 1) (79). Limitation of organ injury and sustained survival by C1 inhibitor therapy has also been reported for a porcine injury model mimicking battlefield injury (110). Further downstream, C3 deficient mice with bilateral femur fracture and hemorrhagic shock resulted in protective effects with reduced circulating DAMPs (e.g., dsDNA), decreased systemic inflammatory response and improved organ performance (e.g., liver enzymes) (111). In a rat model of hemorrhagic shock, C3 depletion by CVF, and the soluble form of CR1 to inhibit C3 action restored vascular reactivity to norepinephrine in the superior mesenteric artery (112). In the clinical setting, C3 inhibition has not been tested in hemorrhagic shock so far except in a recent study from our group, where non-human primates were modeled with severe hemorrhagic shock of 30 mmHg MAP for 1 h, and a delayed application of CP40 was tested (78). C3 blockade by the compstatin compound CP40 seems most promising since there was evidence of improved renal function, attenuated intestinal edema and reduced signs of systemic inflammation and coagulopathy (78). However, the transfer into clinical reality is pending although C3 inhibition seems rather safe in the case of hemorrhagic shock. Most likely, an early inhibition to avoid excessive C3 activation might be enough to improve the outcome (113).

On a C5 level, intestinal injury caused by hypovolemic shock in mice was ameliorated by application of a small peptide C5aR1 antagonist and C5 deficient littermates (114). Similarly, in a rodent model of ruptured aortic abdominal aneurysma with shock, C5 blockade by an anti-C5 antibody revealed some protective effects on remote lung injury with improvement of the bronchoalveolar permeability and myeloperoxidase (MPO) concentrations reflecting reduced neutrophil recruitment and activation (115). Combined inhibition of the complement component C5 and the Toll-like receptor co-factor CD14 in a porcine sepsis model, showed beneficial effects in regard to survival, hemodynamic parameters and systemic inflammation including complement activation (Figure 1, Table 1). First preclinical studies in non-human primates indicate appearance of sC5b-9 in the serum of traumatic-hemorrhagic shock and *ex vivo* effectiveness of C5 inhibitors in prevention of hemolysis, e.g., the small, inhibitory peptide RA101348, commercially-available C5 inhibitory antibodies and Quidel's A217 antibody (106). However, a RCT-clinical study with C5 inhibitory strategies in hemorrhagic shock has not been performed so far most likely based on the rareness and uncertain evidence of a great impact on MODS development in the so far performed preclinical non-human primate studies. Therefore, research providing information about the histological and biochemical data on the multi-organ level in non-human primate studies is necessary for final assessment.

### Targeting Complement in Sepsis

In the case of septic shock, targeting the C5a/C5aR axis seems to be of practical importance since it correlated with the disease severity and mortality and showed promising improvements in different sepsis models (Table 1). Concerning complement-targeted therapies in sepsis, application of RA101295, a 2-kDa macrocyclic peptide inhibitor of C5 cleavage, improved organ performance and survival in an *E. coli-*sepsis model of baboons (75) with lessened evidence of coagulopathy and preserved endothelial and barrier functions. Furthermore, the inhibition of C5 cleavage lead to improved histomorphology of lungs, liver, kidneys, spleen, and adrenal glands suggesting improvement of sepsis-induced MODS (75).

In the same sepsis model, systemic blockade of C3 by compstatin also revealed organ protection on multiple levels. Compstatin showed evidence of sepsis-induced coagulopathy and preserved anti-coagulatory features of the endothelium. Furthermore, C3 blockade improved hemodynamics and heart function and biochemical damage markers of the kidney and liver, indicating protective effects in sepsis-induced MODS (5). For blocking the central complement component C3 during development of sepsis-caused MODS, clinical trials might be safe (113) but these trials are pending and require a special focus on the benefits or maladies for mental alterations during sepsis.

In terms of applicability in the clinical setting, it has been recently shown that blocking solely C5 activation by canonical C5-convertase (e.g., by eculizumab) might not be specific enough since other serine proteases such as trypsin or thrombin can still cleave and activate C5. Therefore, a C5a blocking approach e.g., by IFX-1 anti-C5a antibody has been proposed as a targeted approach in local or systemic infection (116, 117). Overall, the specific complement targets for sepsis-induced MODS might be different than for polytrauma- or hemorrhagic shock-caused MODS especially when given early (Figure 1). The complement target might also change along the course of the disease. It is for example feasible, that early after polytrauma or during hemorrhagic shock a specific C3 inhibition is required, whereas later during development of septic complications, C5a inhibition might help against MODS development (Figure 1). And if complete depletion of a specific complement factor occurs, it might be even wise to replace it, which bolsters the importance of monitoring complement for any clinical trial.

In conclusion, MODS is a culmination of highly heterogeneous events which can involve different and complex stages of complement activation, making it nearly impossible to generate a drug addressing MODS generally. More precisely, the most prominent complement pathways and their down-stream signaling pathways involving multiple trauma with or without hemorrhagic shock and septic shock may be addressed in further preclinical studies.

### Clinical Trials

Considering clinical trials of MODS caused by polytrauma, hemorrhagic shock and sepsis, three observational studies addressed complement activation after polytrauma and severe abdominal sepsis (Table 2). One study focused on the complement activation and expression on leukocytes in polytrauma patients revealing increased complement activity early after trauma and an increased shedding of complement receptors from neutrophil surfaces (NTC00710411; Table 2) (2, 62). Another observational study focuses on the molecular danger response after polytrauma. It is still recruiting patients, and the study aims to include up to 1,000 patients in a collective national data bank in collaboration with the Trauma Research Network (NTF) of the German Society for Orthopedics and Trauma (DGOU) and thus to obtain novel insight into the molecular pattern of complement activity following severe multiple injury (NTF-PT; NCT02682550; Table 2).

**Table 2:** Overview of observational clinical trials evaluating complement activation after polytrauma and sepsis.

**Indication**	**Endpoint**	**Participants**	**Outcome**	**References**
Polytrauma	Inflammatory pattern of complement activation and CRegs on leukocytes	60	Significantly increased serum C3a, C5a, and C5b-9 levels; decreased C5aR expression on neutrophils, which inversely correlated with the clinical outcome; significantly enhanced cC5aR levels, correlating with lethality	NCT00710411
Severe abdominal sepsis	Complement C3 depletion and its association with the down-regulated adaptive immunity	75	C3 depletion was connected to poor prognosis; depletion was associated with coagulopathy and aggravated infection during sepsis	NCT01568853
Polytrauma	Danger response to polytrauma	1000	still recruiting participants	NCT02682550

*cC5aR, circulating form of C5aR; CRegs, complement-regulating proteins; CRP, C—reactive protein*.

A further observational study has set its focus on the central complement C3 by investigation of C3 alterations in patients with severe abdominal sepsis. The aim was to evaluate the impact of C3 on patients' prognosis (NCT01568853; Table 2). This study revealed that C3 depletion is associated with a poor prognosis due to dysregulated coagulation and increased susceptibility for infections (118).

In regard to human studies, three interventional trials have been initiated so far (Table 3). One study addressed the inhibition of upstream complement component (C1) by targeting C1 esterase. This mono-centered double-blind randomized placebo-controlled trial (CAESAR; NCT01275976, Table 3) has been designed using a C1-esterase inhibitor in severely injured patients with femur fracture. Although the rationale of a complement blockade on this level is very well founded because it may also reveal beneficial effects by synchronically inhibition of excessive activation of the coagulation pathway (119), the study has been terminated based on the heterogeneity of patients and challenges in recruitment. A further study has addressed the effect of the C1-esterase inhibitor on human endotoxemia by evaluating its effect on inflammation and marker of organ dysfunction (VECTOR; NCT00785018, Table 3). Detailed results of the study are still pending.

**Table 3:** Overview of complement therapeutics for clinical trials.

**Target**	**Indication**	**Primary endpoint**	**Participants**	**References**
C1 esterase	Trauma or sepsis	Measurement of C1-inhibitor levels, complement concentration and activity, and cytokines; Analysis of neutrophil phenotype and hemodynamic response	Terminated: Study showed limited feasibility	NCT01275976
C1 esterase	Endotoxemia Inflammation MODS	Measurement of C1-inhibitor levels, complement concentration and activity, Cytokines, and markers of inflammation; Analysis of neutrophil phenotype, and hemodynamic response; Assessment of renal injury	20	NCT00785018
C5a	Severe sepsis Septic shock	Evaluation of pharmacodynamic (PD) effects of the C5a antibody	72	NCT02246595

Another clinical trial has been designed and performed to study complement inhibition in early, newly developing septic organ dysfunction (SCIENS; NCT02246595, Table 3) applying a monoclonal antibody against C5a, though the detailed results of this trial have not been published so far. Regarding one cardinal clinical sign of sepsis, the occult or evident alterations of the mental status, inhibitory strategies against C5a might reveal “Janus faced” effects (120). On one hand, C5a inhibition could improve sepsis-impaired blood-brain-barrier, on the other hand, neuroprotective effects by C5a might be compromised (120, 121). Therefore, alterations of the mental status need to be carefully addressed and monitored in any clinical trial using C5a inhibitory strategies.

It is important to note, that it is in general rather difficult to perform interventional studies on polytrauma patients since an informed written consent cannot be provided and the legal representatives are usually difficult to determine within the first hours after severe injury. Therefore, innovative studies early after polytrauma addressing the complement cascade are rather rare and have not been performed on the C3 or C5 level yet.

## Clinical Perspectives

Although various clinically relevant models of trauma, hemorrhagic shock and sepsis have been tested already in non-human primates for the benefit of complement interventions (75, 78, 106), clinical trials in these multidimensional pathophysiologic conditions remain rare (119, 122). When complement intervention strategies are designed for the clinics, the targeted complement factor or activation product needs to be measured before the therapy can be applied. Especially when complex intensive care is necessary which can alter complement levels within a short time period, e.g., by infusion of blood products which contain highly variable concentrations of complement (activation) factors (123) or by extra-corporal circulation devices with large artificial surfaces which may deplete key complement components (124, 125), the exact status of complement activation needs to be determined. This would allow precise and timely intervention either by inhibiting or supporting the complement response after trauma or during sepsis in order to rebalance the immune response. Whereas, various highly effective and specific complement intervention strategies have been developed within the last two decades and are available now (10), in the context of the complex immune response after trauma, hemorrhagic shock and sepsis (9, 126), specific organ damage and function assessment including the immune function at bedside seems far beyond. Therefore, functional monitoring of the organ and immune systems can be considered as a prerequisite before complement interventions move into clinical routine in diseases with a complex pathophysiology. In conclusion, further scientific knowledge and translational efforts are demanded for targeting complement pathways in the setting of trauma, hemorrhagic shock and sepsis with the aim to offer causal therapy and improved outcome.

## Author Contributions

EK and MH-L wrote the manuscript. All authors edited and commented on the manuscript. All authors read and approved the final manuscript.

### Conflict of Interest Statement

JL is the founder of Amyndas Pharmaceuticals, which is developing complement inhibitors (including third-generation compstatin analogs, such as AMY-101) and is the inventor of patents or patent applications that describe the use of complement inhibitors for therapeutic purposes, some of which are developed by Amyndas Pharmaceuticals. JL is also the inventor of the compstatin technology licensed to Apellis Pharmaceuticals [i.e., 4(1MeW)7W/POT-4/APL-1 and PEGylated derivatives]. The remaining authors declare that the research was conducted in the absence of any commercial or financial relationships that could be construed as a potential conflict of interest.

## Abbreviations

CReg: complement regulator
DAMP: danger-associated molecular pattern
HMOX1: heme-oxygenase-1
HS: hemorrhagic shock
MAC: membrane attack complex
MODS: multiple organ dysfunction
MV: microvesicle
NHP: non-human primate
PAMP: pathogen-associated molecular patterns
PICS: persistent inflammation, immunosuppression and catabolism syndrome
PICS: persistent inflammation, immunosuppression and catabolism syndrome
PMN: polymorphonuclear leukocyte
sCR2: soluble complement receptor 2
TNF: tumor necrosis factor.
