# Corrigendum: Targeting Complement Pathways in Polytrauma- and Sepsis-Induced Multiple-Organ Dysfunction

**DOI:** 10.3389/fimmu.2019.00994

**Published:** 2019-05-03

**Authors:** Ebru Karasu, Bo Nilsson, Jörg Köhl, John D. Lambris, Markus Huber-Lang

**Affiliations:** ^1^Institute for Clinical and Experimental Trauma-Immunology, University Hospital of Ulm, Ulm, Germany; ^2^Department of Immunology, Genetics and Pathology (IGP), Laboratory C5:3, Uppsala University, Uppsala, Sweden; ^3^Institute for Systemic Inflammation Research (ISEF), University of Lübeck, Lübeck, Germany; ^4^Division of Immunobiology, Cincinnati Children's Hospital, Cincinnati, OH, United States; ^5^Department of Pathology & Laboratory Medicine, University of Pennsylvania School of Medicine, Philadelphia, PA, United States

**Keywords:** trauma, sepsis, hemorrhagic shock, MODS, complement activation, complement dysregulation, complement therapeutics, clinical trial

In the original article, we neglected to include a conflict of interest statement of Prof. John D. Lambris.

JL is the founder of Amyndas Pharmaceuticals, which is developing complement inhibitors (including third-generation compstatin analogs, such as AMY-101) and is the inventor of patents or patent applications that describe the use of complement inhibitors for therapeutic purposes, some of which are developed by Amyndas Pharmaceuticals. JL is also the inventor of the compstatin technology licensed to Apellis Pharmaceuticals [i.e., 4(1MeW)7W/POT-4/APL-1 and PEGylated derivatives].

The remaining authors declare that the research was conducted in the absence of any commercial or financial relationships that could be construed as a potential conflict of interest.

The authors apologize for this error and state that this does not change the scientific conclusions of the article in any way. The original article has been updated.

